# Quantifying techno-economic indicators' impact on isolated renewable energy systems

**DOI:** 10.1016/j.isci.2021.102730

**Published:** 2021-06-12

**Authors:** Muhammad Shahzad Javed, Tao Ma, Navid Mousavi, Salman Ahmed, Henrik Lund, Hongxing Yang, Yanjun Dai

**Affiliations:** 1 School of Mechanical Engineering, Shanghai Jiao Tong University, Shanghai 200240, China; 2 Engineering Research Centre of Solar Energy and Refrigeration, Ministry of Education, China; 3 School of Engineering, Edith Cowan University, Joondalup, WA 6027, Australia; 4 Department of Planning, Aalborg University, Rendsburggade 14, 9000 Aalborg, Denmark; 5 Department of Building Services Engineering, The Hong Kong Polytechnic University, Hong Kong, China

**Keywords:** Earth sciences, energy policy, energy engineering, energy systems

## Abstract

Addressing climate change with the rising global energy usage necessitates electricity sector decarbonization by rapidly moving toward flexible and efficient off-grid renewable energy systems (RESs). This paper analyzes the wind and solar micro-grids, with batteries and pumped hydro storage for a robust off-grid RES techno-economic operation, while considering diverse multi-objective optimization cases. This research has considered the RES variable operational losses in the developed methodology and relations between different indicators are evaluated, revealing a basic understanding between them. The results reveal that the reliability index is inversely related to the oversupply index, while directly related to the system self-sufficiency index. The cost of energy is more sensitive to technical indicators rather than the storage cost and so can be used as a primary monetary index. Energy and cost balance analysis showed that 16%–20% of the used energy was drained in RES operational losses, which were usually ignored in previous studies.

## Highlights

• A robust techno-economic design is critical for off-grid renewables
• Storage cost is less sensitive to technical indicators compared with energy costs
• Non-used electricity for considered off-grid cases is 45%–55%
• High initial cost (>80%) is the main hindrance to the wide deployment

## Graphical abstract

**Figure:**
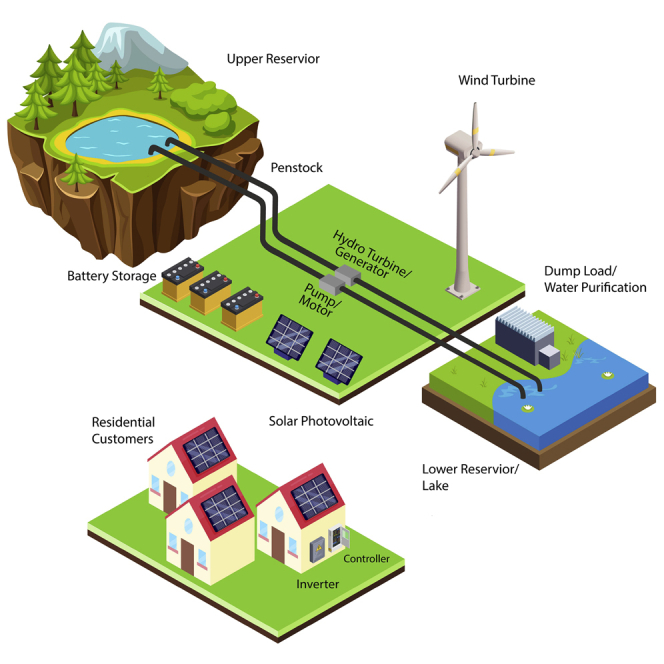

## Introduction

Many countries aim to meet 100% of their electricity demand from renewable or zero-carbon sources by 2040–2050 to meet the Paris agreement goals (Dowling et al., 2020; Rogelj et al., 2016; Mitchell, 2016; Jacobson et al., 2017). Literature studies have seen renewable energy (RE)-based electricity systems as an important and integral part of achieving a fully decarbonized solution (Hansen et al., 2019; Menapace et al., 2020; Connolly et al., 2016; Thellufsen et al., 2020). Meanwhile, intermittent RE integration at a large scale in the national grids may cause serious reliability issues within transmission systems, i.e., blackouts, congestion, and high network impedance (Murdock et al., 2019; Sepulveda et al., 2018; Luo et al., 2015; Khare et al., 2016). The failure of national grids can significantly affect the lives of people living in metropolitan cities and urban areas, where around 3.5 billion people from the global population live and consume two-thirds of the global primary energy that makes 71% of the worldwide greenhouse gas emissions (Perera et al., 2020).

Off-grid renewable energy systems (RES) with electricity storage are crucial to safeguard national grids as large penetration of intermittent RE is out of jurisdiction owing to grids' stability and congestion issues. Meanwhile, some studies suggested the coupling of different energy sectors and continent-wide energy transmission (i.e., Europe) for the high share integration of RE in national grids (Brown et al., 2018). However, these approaches require the agreement of more decision makers and are also not viable for all regions, especially the countries having border disputes. Off-grid RES can also play a significant role in social and economic growth of 1.3 billion people who have no access to electricity, as most of them live in remote areas (Mondiale, 2008; Pimm et al., 2021; Ma and Javed, 2019).

Solar and wind energy are two major pillars of renewable energy resources with the largest (97 GW) and second largest (59 GW) electricity generation capacity in 2019, respectively (Stocks et al., 2020). Moreover, the cost of producing electricity from these technologies has dropped consistently where it is lowest in many regions and is expected to further reduce in the coming decade (Gul et al., 2016; Trancik et al., 2015; Fu et al., 2016; EIA, 2016; Mills and Wiser, 2012; Ziegler et al., 2019; Schmidt et al., 2017). However, for independent solar and wind-based energy systems, some parallel arrangements are required that may include back-up generation, expansion of national grid transmission infrastructure, time-of-use management, and inclusion of appropriate energy storage system (ESS) (Denholm and Margolis, 2007a, 2007b; Solomon et al., 2016; Hirth, 2013; Ma et al., 2015; Fyke, 2019; Tong et al., 2020; Ahmed et al., 2021). These can substantially affect the energy supplying cost, especially for off-grid RES. Moreover, energy system models have been developed with the focus on taking a smart energy system cross-sectoral approach to the analysis of ESS (Chang et al., 2021; Lund et al., 2021). Recent literature studies on national and global levels have identified that EES will be vital to increase the RE penetration either in the grid connected mode or the off-grid mode (Jacobson et al., 2017; Blakers et al., 2017; Esteban et al., 2012). Besides that, the utilization of ESS with off-grid RES becomes more favorable to the areas that are very far from national grids and grid extension is not viable due to the terrain issues or grid transmission infrastructure costs (Denholm and Margolis, 2007a; Hemmati and Saboori, 2016; Luo et al., 2015; Evans et al., 2012). Some studies proposed the diesel generator, a back-up generation source for off-grid RES, as a cost-effective and reliable solution for off-grid RES. However, this solution is not in line with the electricity sector's decarbonization goals (Sundararagavan and Baker, 2012; Braff et al., 2016; Kousksou et al., 2014).

For off-grid RES, the most often used ESS is battery storage (BS)—for small scale—and pumped hydro storage (PHS)—for large scale—owing to their maturity level and low levelized cost of storing energy compared with other available ESS options (Yang and Jackson, 2011; Zhang et al., 2018; May et al., 2018; Schoenung and Hassenzahl, 2003; Krishnakumar et al., 2019; Stocks et al., 2020). PHS has recently emerged and is seen as a benign option for the assessment of energy mix options with the high penetration of low-carbon sources. A recent study investigated the global off-river locations for PHS and identified 616,000 potential locations, revealing the available excessive storage options to exploit the solar and wind potential, especially for the remote sites (Stocks et al., 2020). On the other hand, many literature studies have proposed BS for RES owing to its advantages over other ESS such as high efficiency, fast response time, and scaling feasibility due to modular structure (Hesse et al., 2017). However, these ESS have rarely been explored as hybrid storage for the off-grid RES, which can significantly enhance the system's reliability level owing to the complementary characteristics between them (Javed et al., 2020; Abdelshafy et al., 2020). Many literature studies have comprehensively reviewed the weaknesses, strengths, and application of the BS and PHS regarding the RE environment (Beaudin et al., 2015; Kocaman and Modi, 2017; Ibrahim et al., 2008; Javed et al., 2021; Guezgouz et al., 2019a, 2019b; Abdelshafy et al., 2020; Jurasz et al., 2020). For example, BS has a high response time (Luo et al., 2015); it can be used to cover the instantaneous and small demand-supply gaps until PHS starts operation with constant output voltage, which is due to the PHS lag time. Furthermore, BS would cover small demand-supply gaps, whereas PHS would be used to manage large deficits (and surplus); thus, low-efficiency PHS power output can be avoided at partial loads, i.e., when RE directly covers a portion of the baseload (Javed et al., 2020).

The methodologies proposed in the literature for off-grid RES may notably lead to reliability issues since previous studies disregarded the ESS operational losses by either ignoring or considering as constant (Ma et al., 2014). The optimal sizing that is performed while considering these approaches may not be robust, and the designed RES would not meet the outlined objectives, especially when RE sources are being used as base energy source, i.e., off-grid RES. Moreover, previous optimization studies often only considered one aspect at the design stage, either technical or economic, that would lead to the issues like loss of load or high initial capital cost, which are the major obstacles in the widespread propagation of off-grid RES. Considering the aforementioned issues, in this study we first extended an experientially verified PHS model (Mousavi et al., 2019) that considered all types of hydraulic, mechanical, electrical, and efficiency losses, by integrating a BS and RE generators model to effectively evaluate the techno-economic performance of hybrid storage-based off-grid RES that has not been documented before. Then, the developed mathematical model with the proposed energy management strategy (EMS) is assessed considering different system evaluation indicators. Furthermore, different multi-objective optimization cases are developed to ensure robust capacity sizing and RES operation to alleviate RE sources' vulnerability.

The robust techno-economic capacity sizing based on the developed EMS for off-grid RES is indispensable. Considering the intermittent RE as a base source for off-grid RES and high initial investment cost, robust optimal sizing is indispensable that considers all types of losses and system evaluation indicators like RE self-sufficiency, reliability level, oversupply index, and ESS cost. This study developed a methodology for operational and economic analysis of off-grid RES, focusing on considering all operating losses and various RES evaluation indicators simultaneously. We investigated the relationship between technical and economic RES evaluation indicators, their comparative tendency in multi-objective optimization, and their impact on the off-grid RES performance.

This study provides a basis for designing and deploying a robust off-grid RES with storage by keeping in view the defined technical (reliability level, self-sufficiency, oversupply) and economic (storage cost and cost of energy) project objectives. To the authors' knowledge, there are very rare studies that systematically explore the techno-economic feasibility of off-grid RES and provide a path to achieve the decarbonization goals in the electricity sector. The robust and comprehensive findings may increase the confidence regarding off-grid RES deployment, especially remote places where extension of national grids is either expensive or out of the jurisdiction. Moreover, off-grid RES can ensure affordable and reliable energy provision to 1.3 billion people who have no access to electricity owing to lack of electricity infrastructure, especially in developing regions like India and sub-Saharan Africa.

## Results

We have presented the results in three subsections. At first, we discuss the diverse bi-objective, tri-objective optimization cases considering different reliability levels, and the objectives (technical-economic, economic-economic, and technical-technical) relationships are assessed. Then, off-grid RES energy and cost-share analysis at different reliability levels is accomplished. It provides operators and researchers a way to analyze the newly installed energy systems where blackouts are not an option. After that, the effect of operational losses, i.e., mechanical, electrical, efficiency, and hydraulic, is visualized to ensure the reliable working of hybrid storage based off-grid RES by developing a generalized methodology that has not been documented before. This is followed by the method details used in this study.

### Multi-objective optimization of RES considering different set of objectives

Several previous studies focused on optimizing off-grid RES; however, those studies considered either a limited number of objectives (one or two) or limited RES components (one source with one storage). Moreover, results from those studies did not provide a framework for investors/policymakers to select appropriate objectives for robust optimization of RES, especially when RES is off-grid and significant initial investment is involved. We considered several cases for a range of objectives (both technical and economic), and multi-objective optimization is performed to assess the synergies between different objectives. All cases are simulated considering 90% and 95% reliability level assuming that 5%–10% load is a part of the demand side management and can be taken care by strategies like time of use.

**Figure 1:**
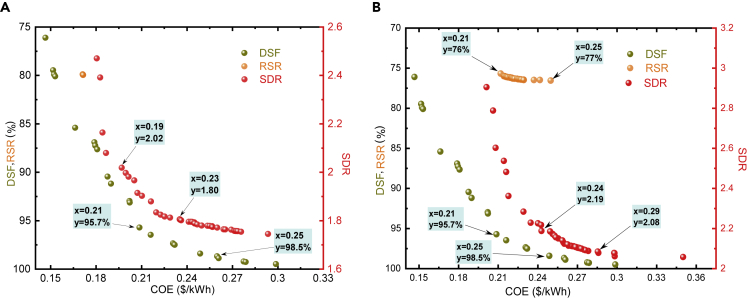
RES optimization with two objectives at different reliability levels (A) 90% (B) 95% DSF, demand-supply fraction; RSR, ratio of energy directly supplied by RE to RES to cover load; SDR, oversupply index. The definitions of optimization objectives are provided in the supplemental information (see Section S5). The left vertical axis represents the two objectives (DSF, RSR), and the right vertical column contains SDR objective value. The figures show that there is a set of non-dominated solutions for each multi-objective optimization case. There is a trade-off zone (represented with arrows) where decision makers have to compromise between the cost of energy (COE) and defined project objectives. These trade-off areas are selected based on the percent increment in COE with respect to the respective objective value and reflect the least percent increment in COE value concerning the specified reliability level. The reliability level constraint is only set for RSR and SDR cases, as DSF is an index for reliability and thus its compromise zone is the same for both scenarios. The effect of different reliability levels on SDR and RSR is evident from figures and it can be noted that the compromise zone shifted to high oversupply (2.02–2.19) and high COE (0.19–0.24 $/kWh), as the reliability level of RES changed from 90% to 95%. In the COE-RSR case, the optimizer found a limited number of non-dominated solutions in each scenario, which shows the importance of ESS in off-grid RES. It also highlights that, despite employing more than one RE source, energy directly supplied by RE generators to cover the load demand is not enough to meet the required reliability level, i.e., the value of RSR is 80% for 90% DSF and 76%–77% for 95% DSF, illustrating the consideration of ESS to mitigate the disharmony between demand-supply. Corresponding decision variable values of non-dominated solution sets of all optimization cases are presented in the supplemental spreadsheet: Data S1.

Optimization with two objectives reveals no specific solution (Figure 1). There is always a trade-off zone with a set of solutions; however, a compromise can be made based on the defined priorities, i.e., level of reliability, maximum investment available (see Videos S1 and S2: Demonstration of multi-objective optimization simulation and Demonstration of exploration and exploitation phases in multi-objective optimization). Furthermore, the results can be altered by forcing the optimizer toward a particular direction (see optimizer description—Section S4). For instance, a remote place is too hot, where high ESS capacity is not desirable—batteries performance degrade at high temperatures and water will evaporate exponentially from PHS reservoirs—the lower and upper bounds of ESS decision variable can be changed accordingly by the operator, and optimizer will provide a set of non-dominated solutions that contain high RSR with the least cost of energy (COE) for the same reliability level (see Figure S6 for optimizer application with developed EMS). In multi-objective optimization, non-dominated solutions, also called Pareto front, refer to a set of solutions across the feasible region of the design variables that are not dominated by any other solution set during optimization. It allows decision makers to anticipate the accurate approximation and select one of the obtained solutions based on the defined objectives. It is worth noting that the oversupply index (SDR) has a high value, even though the two RE sources and two ESS are employed, revealing that off-grid RES has to be oversized up to a specific limit due to RE variability, disharmony between demand and supply, and to prevent blackouts. The oversupply index value in our study (Figure 1A: 1.8–2.2 and see Data S1) is higher than in previous literature studies (Shabani et al., 2020) (1.3–1.5). It is because we considered all types of losses that occurred during the RES operation, and the optimizer makes sure to have enough energy to satisfy both energy losses (see Figure 3) and load demand.

Document S1. Figures S1–S9 and Tables S1–S7

Data S1. Optimization with two objectives, related to figure 1

**Figure 2:**
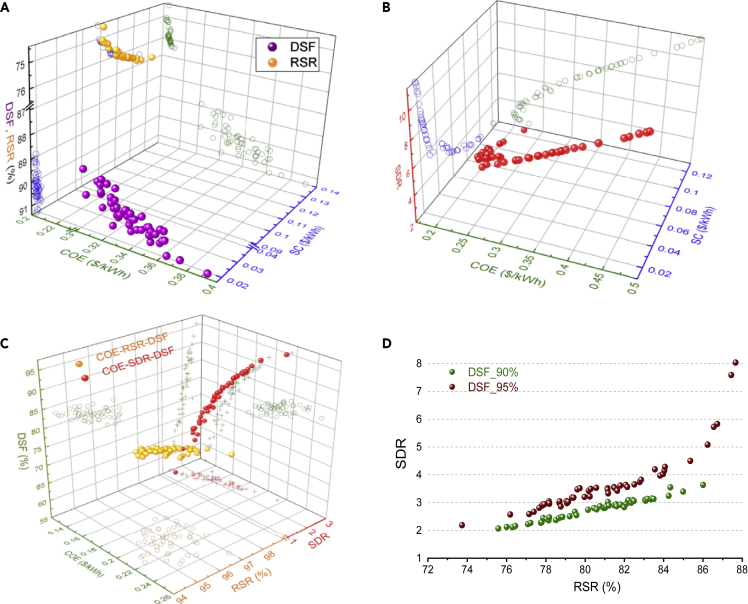
RES optimization with three objectives at reliability level of 95% (A) In this scenario, each case considered the two economic objectives (storage cost and COE) and one technical objective. There is no reliability constraint for the cases that contain the DSF as the optimization objective. XZ, YZ projections show the direct relationship of the respective two objectives seeing the third objective value. The figure illustrates that COE is more sensitive to DSF than the SC, revealing that RE generators' appropriate size is essential to achieve the required reliability level in the off-grid RES. Similarly, no significant changes are observed in RSR with the increment in SC value; however, it is susceptible to COE illustrating policymakers' role in defining the priorities for robust optimization to gain the predefined project goals. (B) This figure shows that the oversupply index (SDR) has an inverse relation with SC and direct with COE. (C) In this scenario, each case considered the one economic objective (COE) and two technical objectives. The figure shows that RSR and DSF have an inverse relationship and optimizer preferred to compromise the system reliability compared with the RSR and lessened the system COE value significantly, revealing that adding RE generators capacity is more economical than the ESS, which is in line with the findings of Figure 2A. This figure also demonstrates the SDR and DSF proportional relationship. (D) Optimization results of COE-SDR-RSR are shown in this figure. The SDR and RSR relationship at different reliability levels is represented in this figure, whereas their relationship with COE can be observed in Figures 2A and 2C. The decision variable values of non-dominated solution sets of all cases are presented in the supplemental spreadsheet: Data S2. Also see Video S2 (demonstration of multi-objective optimization simulation) to comprehend the presented multi-objective optimization results. DSF, demand-supply fraction; RSR, ratio of energy directly supplied by RE to RES to cover load; SDR, oversupply index; COE, cost of energy; SC, storage cost (for detailed description of indicators, see supplemental information Section S5).

**Figure 3:**
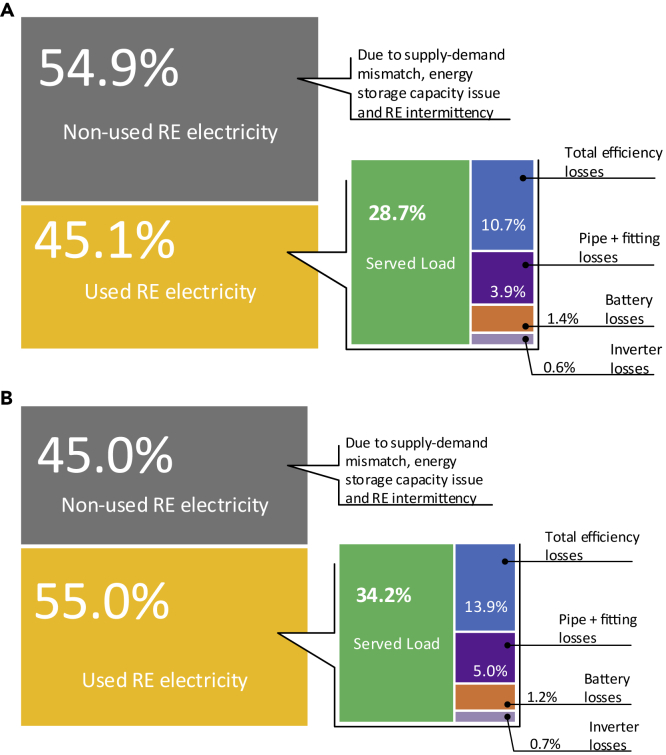
Energy share analysis of total energy produced by RE generators at different reliability levels (A) 95% (B) 90% The figures illustrate the division of total energy produced by RE generators for 1 year. The mathematical modeling of RES electricity generation components is presented in the supplemental information (see Section S2), WHILE for ESS and losses modeling see method details. The analysis assumed that total energy produced by RE generator components is 100% and then energy consumed during RES operation by each component is calculated separately using developed components and losses models. The input data, i.e., solar irradiance, wind speed, and load design, are presented in the supplemental information (see Section 2). The specifications of configurations used for energy share analysis are shown in Table S1. Configurations are selected from the COE-RSR-SDR case at DSF constraints of 90% and 95% with the least COE value.

Analysis with three objectives shows that two technical indicators (DSF and RSR) are more sensitive to COE compared with the storage cost (SC) (see Figures 2A and 2B and supplemental spreadsheet: Data S2) and reveals that appropriate selection of objective functions for robust optimization is essential. Furthermore, it is also important to note that ESS can play a key role in compromising between the oversupply index and the reliability index. It also reveals that, to date, adding RE generators capacity is economical compared with ESS to satisfy a certain reliability level for off-grid RES (see Figures 2A and 2C). On the other hand, the addition of RE generators capacity leads to high oversupply (see Figure 2D), leading to an increase in overall non-used RE electricity (See Figure 3). The results of tri-objective optimization with a 90% reliability level are presented in the supplemental information (see Figure S1). Recent literature studies show that the cost reduction trend of ESS is expected to continue in the future, and the deployment of off-grid RES will gain upsurge shortly. The results indicate two ways to meet the load demand for off-grid RES to meet the required reliability level. It can be achieved either by an increment in RE generators size or by adding more ESS; however, it cannot be done impulsively and will depend on other indicators defined by policymakers. For instance, if one country's government provides subsidies for the deployment of RE only and high ESS capacity causing the increase in initial capital cost, then a certain reliability level can be achieved by enhancing the range of RSR indicator and SDR indicator can be ignored. Similarly, if there are remote places where available RE sources are not sufficient, consideration of ESS size will be the key to satisfy the certain DSF. However, at the same time, SC and RSR will become essential indicators for robust optimization.

Based on multi-objective optimization cases, we draw relationships between all the considered technical and economic optimization objectives based on the comprehensively performed analysis (see Table 1). This table can provide a deep insight into all designers/policy makers to select optimization objectives because RES problems come up with complex, non-linear, and non-convex objective functions. It will become computationally costly to consider all the cases simultaneously, especially when the system is isolated and many decision variables are involved, i.e., losses, penstock sizes, and RE generators. In such a case, additional results can be drawn seeing the relative behaviors of objective functions. For instance, SDR has an inverse relation with DSF, whereas it behaves linearly with RSR. Similarly, our analysis also revealed that SC is less sensitive to technical indicators than COE; thus, COE can be a primary economic index for robust techno-economic optimization (see Figures 2A and 2C).

**Table 1:** Relationship between objective functions in multi-objective optimization

	COE	DSF	RSR	SDR	SC
COE	–	✓	✓	✓^a^	--b
DSF	–	–	✕^c^	✓	✓
RSR	–	–	–	✓	✓
SDR	–	–	–	–	✕✕

✕, inverse; ✕✕, strong inverse; ✓, linear.

DSF, demand-supply fraction; RSR, the ratio of energy directly supplied by RE to RES to cover load; SDR, oversupply index; COE, cost of energy; SC, storage cost.

a Inverse relationship between COE and SDR is observed in two objective optimizations; however, when the system is optimized considering three objectives, COE and SDR reflect strong linear behavior.

b The relationship between SC and COE cannot be defined as it depends on many variables simultaneously, i.e., RE generators cost and capacity, level of system reliability.

c RSR and DSF are linearly proportional to a specific limit, i.e., at very low DSF, which is always undesirable.

### Energy and cost balance for off-grid renewable energy system

**Figure 4:**
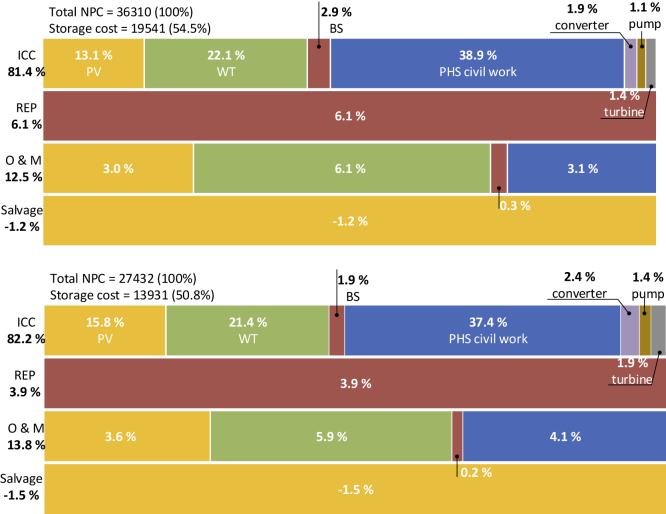
Cost share analysis of an off-grid RES at different reliability levels (A) 95% (B) 90% The figures show the division of cost incurred on the off-grid RES during the whole project lifetime (20 years in this study). All future cash flows and expenditures are converted into the net present cost (NPC) using a discount rate concept (Javed et al., 2021). The economic parameters of RES components are provided in the supplemental information. All costs presented are in USD. Total RES cost (NPC) is considered 100% and then each type of cost expense of RES components is simulated in MATLAB. The percent of storage cost represents its share in the total NPC used for ESS. Each color of the figures represents a specific RES component, and each row illustrates one type of cost. The last row shows the salvage value with a negative sign, representing positive cash flow and subtracted from NPC at the end of the project lifetime. Specification of the configurations used for economic analysis is the same as used for energy share analysis (see Table S1). ICC, initial capital cost; REP, replacement cost; O&M, operation and maintenance cost; PV, photovoltaic; WT, wind turbine; BS, battery storage; PHS, pumped hydro storage.

We next performed a detailed energy share analysis of a techno-economically optimized configuration at different reliability levels. It reveals that there is still a significant amount of non-used electricity (see Figure 3). The energy analysis unveils that RE sources' variable nature will remain challenging to off-grid RES, even though the RES is precisely modeled—considering all operation losses—and optimized (considering all techno-economic indicators). As the reliability level increases, the share of non-used electricity rises and vice versa. It also reflects that the DSF indicator is key at the design stage of off-grid RES, which affects not only the size of RE generators (see Table S1) but cost as well (see Figure 4). Meanwhile, DSF cannot be varied impulsively, and its relation with other RES evaluation indicators must be considered. For instance, it may increase SC significantly and eventually leads the RES to be more dependent on ESS. However, sometimes there are periods when RE is not available for more extended periods, and the system may face blackouts/shutdown that is the point where RSR comes in. However, literature studies have shown that wasted RE energy due to curtailment can be reduced to 0%–14% by deploying RES with firm low carbon sources (Sepulveda et al., 2018).

Energy analysis also reveals that a significant amount of energy is consumed to cover the system losses during operation that are either ignored or considered constant in previous studies, particularly those that performed the optimization and techno-economic analysis for off-grid RES. In both cases—either losses are ignored or considered as constant—capacity sizing of RES components may not be robust, and deployed RES may not perform at the required reliability level, thus can affect the growing deployments of RES to meet the goal of deep carbonization of the electricity sector. Notable changes occurred in losses at different reliability levels, which shows setting constant value is firmly inappropriate (see Figure 3). For example, PHS losses are high at a lower reliability level (90%)—increased from 14.6% to 18.9%—because pump/turbine machine efficiency degrades when it operates at partial loads (see supplemental information, Section S6, data provided by manufacturers). At the same time, this is not the case in battery storage, and losses decreased with reliability level. The developers/designers can perform this type of detailed energy analysis for on-site working RES or newly designed RES, and robust operation of off-grid RES can be assured. Moreover, this energy analysis can be replicated for other RES configurations; for instance, if only one RE source or ESS is there, other components can be removed from modeling and vice versa.

We also analyzed each RES component's cost share in the total net present cost (NPC) incurred on off-grid RES during the whole project lifetime (Figure 4). The detailed investigation reveals that a major part of the RES total cost comprises the initial capital cost (ICC) and is a major hindrance for the deployment of off-grid RES. In both cases, more than 80% of total RES expenses are incurred in the form of ICC, which reveals the critical role of governments/organizations that certain level of subsidies should be provided to enhance the off-grid RES deployment and thus meet the deep decarbonization electricity sector goal. Cost analysis also illustrates that more than 50% of the total NPC is used to cover the SC and makes the deployment of small isolated RES infeasible. However, different low carbon power supply options can be considered initially with off-grid RES that can substantially reduce the ICC and increase the demand-supply flexibility (Ma et al., 2015). The inclusion of firm low-carbon resources—these resources can be varied depending on the region, available resources, and local government policies—will substitute ESS for the specified time. Hence, it may allow authorities to integrate ESS stepwise. This approach can considerably increase the share of off-grid RES in the electricity sector without influencing the national grids, i.e., congestion, and finally, the utilization rate of carbon-free energy resources will upsurge.

### Working presentation of developed methodology to evaluate the robustness of designed renewable energy system

It is a fact that RE sources—especially wind and solar due to mature technology and least expensive among available RE—will be the key to decarbonizing the electricity sector. However, RE sources' intermittent nature is the main hindrance to the widespread application, especially when they have to use as a base source, i.e., in remote places or off-grid. Therefore, a firm operating strategy—not only at the control level of RES but also at the individual component level—for the robust RES operation should be developed to alleviate the heavy dependence of the electricity sector on fossil fuel-based energy, especially remote places where most of the energy needs are still met either using diesel generators only or as back up with RE sources. First, we developed a detailed and comprehensive mathematical model for hybrid storage (see method details)—battery and PHS are mature and recommended storage for off-grid RES (Guezgouz et al., 2019b; Javed et al., 2020)—and integrated with wind and solar model (see supplemental information, Section S2). To validate the performance of the developed mathematical model, we gathered the technical and economic details of all RES components from manufacturers (see supplemental information) and simulated the optimized configuration, at a reliability level of 95% (Table S1), for a designed small load of 1 year (Figure S3). Furthermore, all types of losses incurred during the operation of RES, including ESS losses, are considered, making the performed simulation more realistic and providing a framework for designers to validate the optimized RES before placing significant investments. Such compact mathematical modeling of RES will assure the RES working at designed objectives, enhance the investors' confidence, and thus increase the deployment of off-grid RES. The proposed mathematical model can be imitated at any scale and any type of isolated RES configuration, i.e., solar-battery, solar-PHS, and wind-battery-PHS.

**Figure 5:**
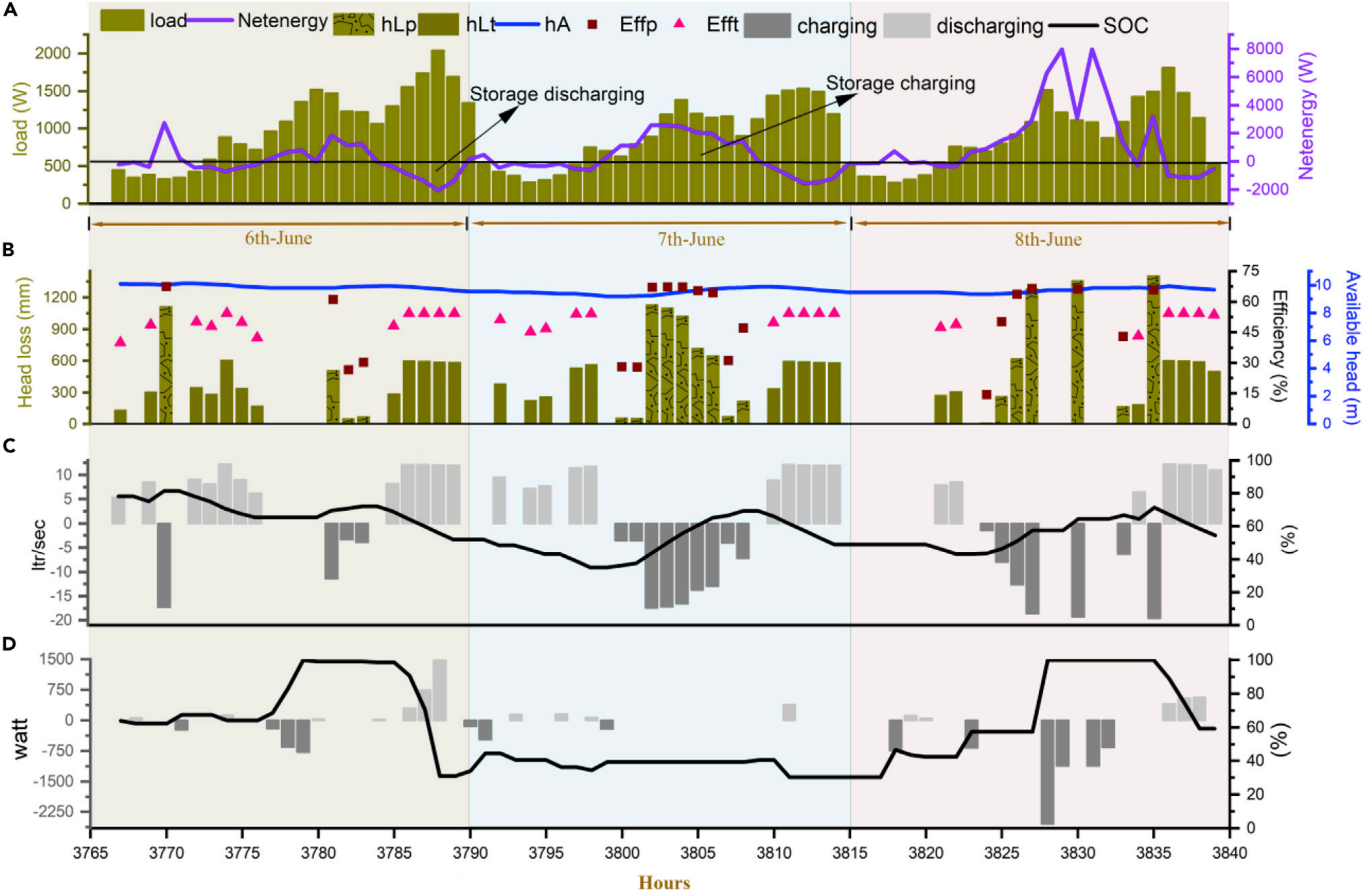
Working presentation of the developed methodology for an off-grid RES The figures illustrate the RES operation during three consecutive days in June. The proposed working presentation is based on the developed methodology and EMS (see method details) that considered all types of losses, i.e., mechanical, electrical, hydraulic, and efficiency, to make simulation results more realistic. The specifications of the RES configuration selected for simulation are presented in Table S1 (with 95% reliability level). The PHS model employed in this study is experimentally verified and substantially extended for integration with proposed RES. (A) Figure shows the load demand (based on Figure S3), net energy, and ESS charging/discharging periods during the mentioned days. (B) Efficiency variations in pump/turbine at partial loads (for the data provided by the manufacturers, see supplemental information, Section S6) are visible in the figure that often has been ignored or assumed constant in literature studies. The left vertical axis represents the head losses for pump/turbine due to the penstock and fittings (see method details). The actual available head (see supplemental information for the description of different PHS head terms, Section S3.1) and the efficiency of the pump/turbine are shown on the right vertical column. (C) Continuous charging and discharging periods, variation in water flow rate for available net energy and state of charge (SOC) of the PHS are illustrated in this figure. (D) Battery storage is used as supplementary storage in the proposed EMS, and its role to cover the small shortages and consume small surplus's (see method details) is visible in the figure. hLp, pump head loss; hLt, hydro turbine head loss; Effp, pump efficiency; Efft, turbine efficiency.

The complementary functionality of PHS and battery storage is visible from the working presentation (Figures 5C and 5D) and reveals the effectiveness of hybrid storage for robust operation of off-grid RES. For example, battery storage recharged when a small amount of net energy was available (between 3,775 and 3,780 h) and then for the following hours (3,780–3,785 h), the controller drives the PHS due to the high net surplus energy. Furthermore, considerable variation in pump/turbine machines efficiency and head losses can be observed with respect to the available net energy and available head that makes the proposed model and simulation more realistic (Figure 5B). It is important to note that most of the literature studies assumed the available head for pump/turbine as constant by saying that the reservoir surface area is big enough; however, this cannot be the real case always, for instance, closed-loop PHS for small village/community. About 10.7% of the total served energy consumed to meet the RES efficiency losses are often disregarded in the literature techno-economic studies (Figure 3A). Moreover, head losses due to pipes and fittings are also shown in Figure 5B that accounts for 3.9% of the served energy (see Figure 3A). The PHS models used in literature studies for feasibility and techno-economic optimization often overlooked them that may undermine the optimal sizing of RES (Makhdoomi and Askarzadeh, 2020; Simão and Ramos, 2020; Al-Ghussain et al., 2020; Bhayo et al., 2020).

**Figure 6:**
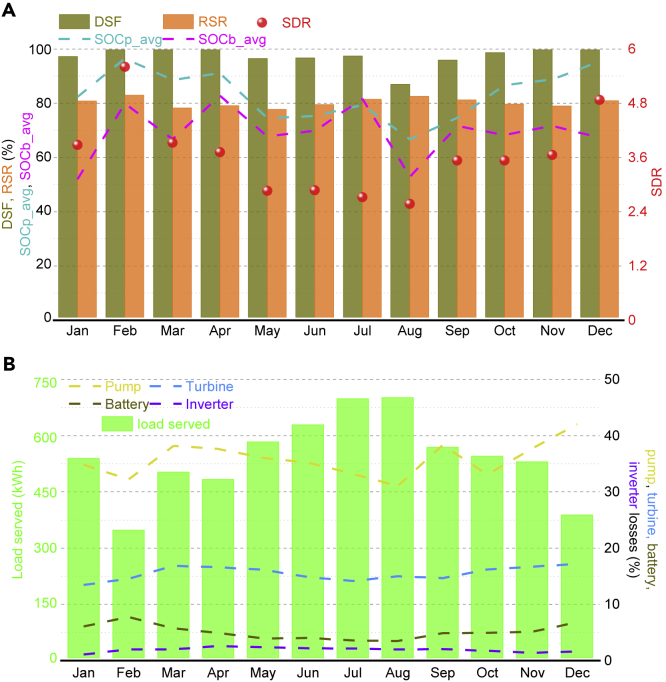
Monthly average variations in objective values and losses of RES at reliability level of 95% The figures illustrate the RES performance to assess the optimized RES against defined objectives. Specifications of the configuration used for this analysis are presented in Table S1. The developed methodology is simulated in MATLAB for a whole year. (A) Figure shows the monthly average values of RES evaluation indicators and SOC of ESS. The left vertical axis represents all values in terms of percentage, and only SDR values are presented on the right vertical axis. The average value of DSF is more than 95% for the entire year except August owing to peak summer load during that month. However, the whole year average DSF is more than 95% that satisfies the designed reliability objective, i.e., 95%. No significant changes are observed in RSR average value throughout the year that illustrates the significance of ESS for off-grid energy systems to achieve the required reliability level. The average SOC of PHS lay between 60% and 100% during the whole year, whereas the average SOC of battery lay between 55% and 80%, revealing that the designed RES is robust and substantially met the designed RES objectives. It is worthwhile to note that RES has the least oversupply in summer owing to the high load demand (See Figure S3) and vice versa. (B) Figure illustrates the load served by RES during each month and how much percent of the served load is consumed to satisfy the RES operation losses. The pump and turbine machine losses include penstock losses, efficiency losses, and motor/generator losses (see method details). Notable variations in all types of losses throughout the year are visible, making the simulation results more realistic and practical. DSF, demand supply fraction; RSR, ratio of energy supplied by RE to total energy supplied by the system; SDR, oversupply index; SOCp_avg, average SOC of PHS; SOCb_avg, average battery storage SOC (for definition of all indicators, see supplemental information, Section S5).

Finally, we performed a 1-year simulation to assess the optimal design of RES, keeping in view the evaluation indicators used as optimization objectives (Figure 6). Figure 6A also verifies the relation between technical objectives that are drawn in Table1. For example, in August DSF has the least value, but at the same time, SDR is also at a minimum level, revealing the linear relationship between them. Meanwhile, for the same month, RSR is high, which reflects the inverse relation with DSF. Designed RES has the lowest demand in February and peak energy demand in August due to added seasonal randomness (Figure S3), where the techno-economically optimized RES effectively covers the substantial demand-supply gap without violating any defined system objectives range beside that all type of losses were also designed as a variable. Overall, the proposed framework effectively covers how to set design objectives and their range, relation between different technical and economic evaluation indicators, how to model an off-grid RES with operational losses, how to assess the techno-economic performance of an off-grid RES comparatively, and, finally, the evaluation of the robustness of an off-grid RES to safeguard the investors' investment.

## Discussion

Off-grid RES is essential for many reasons: reduce the increasing grid stability concerns due to the large integration of variable RE, deeply decarbonize the electricity sector to mitigate climate change, and improve the economic and living conditions of remote areas. For the efficient working of off-grid RES, ESS is crucial to alleviate the RE intermittency (Arent et al., 2020) and demand-supply gap and achieve a certain reliability level and monetary benefits in the long run. The methods and strategies about the cost-containing of off-grid RES are frequently presented in previous studies; however, their proposed models were not comprehensive and did not account for all types of losses incurred during the operation of RES that questions the robustness of their proposed solutions. In the proposed study, we extended an experimentally verified PHS model and a battery storage model is integrated along with RE generators and small designed load, to evaluate the relation between different RES evaluation indicators—technical (reliability, RE self-sufficiency, over-supply index) and economic (cost of energy, storage cost)—and provide a framework for the investors and policymakers regarding the deployment of off-grid RES. The proposed framework also provides a mechanism and idea to designers about selecting a different set of evaluation indicators (technical and economic) for multi-objective optimization and then how to assess the optimally designed configuration in terms of useful energy, energy used to cover losses, and unused energy.

We analyzed the diverse multi-objective optimization cases by considering the different sets of technical and economic indicators simultaneously. Relationships between them are derived to provide an idea for the robust design of off-grid RES. The most often developed RES optimization problems are non-linear and involve several decision variables that make the optimization more complicated, computationally expensive, and time-consuming. The relationships derived in this study between technical and economic objectives can help designers to select appropriate optimization objectives and obtain a robust configuration without optimizing the RES repetitively considering the different set of objectives each time. It means that RES can be optimized considering one or two objectives, and the trend of other objectives can be estimated.

Furthermore, the proposed relationships are developed considering a comprehensive mathematical model based on off-grid RES that considered all types of losses (electrical, mechanical, efficiency, and hydraulic losses) and ensures the developed relationships' robustness. The PHS and battery storage are employed for this study because these storages have been repetitively used in the literature for the off-grid RES and are mature technologies compared with other available ESS. Moreover, these two storages have the highest share of the total installed worldwide energy storage capacity (Javed et al., 2019a). The roadmap proposed in this study can also be followed to comparatively evaluate the different sets of configurations and select the best RES configuration for remote/off-grid places in terms of useful energy, energy consumed for losses, non-used energy, and techno-economic performance, seeing the preset project objectives.

A thorough techno-economic studied and optimized RES is critical to achieve deep decarbonization in the electricity sector, as energy systems are long-lived assets and installed capacity during the next decade is likely to persist until 2050. Recognition of the off-grid RES—to increase the penetration of RE and reduce the cost of deep carbonization—has instant implications and requires an inclusive future planning of electric power systems to meet the climate change mitigation policies and for further advancement in RE and ESS technologies research. Although heavy subsidies and public policies support variable RE's growth, a more compact and reliable EMS for off-grid RES to meet the targeted goals is indispensable; otherwise, the target of deep decarbonization in the electricity sector will be out of jurisdictions. Our results illustrate that different sets of objectives for RES have a significant impact not only on components size (decision variables) but on system evaluation indicators as well, for instance, initial capital cost and oversupply. If the off-grid RES is the need of the day, proposed detailed methodological energy analysis of an optimized system can provide a way to accomplish the goals.

### Limitations of the study

This study can be extended further by considering several aspects in the future. First, a wide range of scenarios by replacing/adding all available energy sources and ESS options—keeping in view the geographical, monetary, and social constraints—can be developed, and location-specific best RES configurations can be attained. Our study presents a pathway to design, model, and simulate the off-grid RES considering all operational losses. Consideration of the cross-sectoral approach (i.e., heating/cooling load demand) is beyond the scope of this study. Still, it can be considered in future studies and the ESS model presented in this work can be used. Second, this work considered a finite set of techno-economic indicators that can be extended/improved to analyze further the off-grid RES role in achieving the carbon neutrality goal. Third, this analysis can be extended for a mix of RE technologies with other low-carbon power generation sources, given that RE generators put forward as the primary source of power, and certain flexibility can be attained to make sure the high system reliability for sensitive off-grid dispatch zones, i.e., commercial zones. Technology mix-specific policies can be considered a short-term option in the way of deep decarbonization of the electricity sector. Fourth, this work considered only 1-year resource data that would not reflect the spatiotemporal and inter-annual RE sources variability. In future work, the presented energy system model is expected to integrate with decadal RE sources data to examine the resource complementarity impact on off-grid RES reliability; thus, robust and absolute dependable zero-carbon power systems can be developed.

## STAR★Methods

### Key resources table

**Table:** 

REAGENT or RESOURCE	SOURCE	IDENTIFIER
**Deposited data**		

Hydraulic parameters of pump/hydro-turbine i.e., head, efficiency-power curve	Mousavi et al.	https://doi.org/10.1016/j.apenergy.2019.03.015
Darcy-Weisbach model for available water-head modelling	Shammas & Wang et al.	Shammas, N. K., & Wang, L. K. (2015). Water engineering: hydraulics, distribution and treatment. John Wiley & Sons.
Technical figures of pump/hydro-turbine	Mousavi et al.	https://doi.org/10.1016/j.apenergy.2019.03.015
PHS reservoirs water volume calculation model	Javed et al.	https://doi.org/10.1016/j.apenergy.2019.114026
Penstock losses modelling parameters	Mousavi et al.	https://doi.org/10.1016/j.apenergy.2019.03.015
Battery storage charging and discharging parameters	Ma et al.	https://doi.org/10.1016/j.enconman.2018.12.059
Technical specification of solar module	Javed et al.	https://doi.org/10.1016/j.energy.2019.03.131
Technical specification of wind turbine	Manufacturer	https://www.windpowercn.com/products/21.html
Irradiance and wind speed input data	Javed et al.	https://doi.org/10.1016/j.apenergy.2019.114026
Cost figures of pumped hydro storage components i.e., pipes, civil work, pump/turbine	Based on local market	N/A
Cost figures of battery storage including O&M cost	Ma et al.	https://doi.org/10.1016/j.apenergy.2014.01.090
Capital and O&M cost of solar module	Guezgouz et al.	https://doi.org/10.1016/j.enconman.2019.112046
Capital and O&M cost of wind turbine	Manufacturer	https://www.windpowercn.com/products/21.html
Data for synthetic design of load demand	Javed et al.	https://doi.org/10.1016/j.energy.2019.03.131
Hourly and daily added randomness in load profile	Javed et al.	https://doi.org/10.1016/j.apenergy.2019.114026

**Software and Algorithms**		

Grey wolf optimizer algorithm	Mirjalili et al.	https://doi.org/10.1016/j.advengsoft.2013.12.007
Matlab	MathWorks	https://www.mathworks.com

### Resource availability

#### Lead contact

Further information and requests for resources should be directed to and will be fulfilled by the Lead Contact, Tao Ma (tao.ma@connect.polyu.hk)

#### Materials availability

This study did not generate new unique reagents.

#### Data and code availability

The M-script files are available for academic purposes upon reasonable request.

### Method details

#### Nomenclature

The specifications of all symbols used below in energy system modeling are presented in the following table.

**Table:** 

Symbol	Definition
Abbreviations	
*a*_*t*_	area of hydro turbine pipe (m^2^)
*α*_*PV*_	temperature coefficient of power
*AC*	annual cost ($)
*BS*_*c*_	available charging power for BS
*BS*_*d*_	power discharged from BS (watt)
*D*	diameter of the penstock (m)
*D*_*p*_	pump pipe diameter (m)
*D*_*t*_	turbine pipe diameter (m)
*F*	friction factor
*E*_*NS*_	total energy not served (kWh)
*G*_*PV*_(*t*)	incident irradiance (kW/m^2^)
*G*_*STC*_	standard irradiance (kW/m^2^)
*G*	acceleration due to gravity (9.8 m/s^2^)
*h*_*a*_	active head (m)
*h*_*lr*_	lower reservoir height (m)
*h*_*lrw*_	lower reservoir water height (m)
*h*_*p*_	available pump head (m)
*h*_*pl*_	pump head loss (m)
*h*_*s*_	vertical distance between LR and UR
*h*_*t*_	available turbine head (m)
	hydro turbine head loss (m)
*h*_*ur*_	upper reservoir height (m)
*h*_*urw*_	height of water in upper reservoir (m)
*I*_*b*_	nominal battery current (ampere)
*IC*	initial capital cost ($)
*K*_*fittings*_	resistance coefficient of fittings
*L*	length of the penstock (m)
*L*_*p*_	pump pipe length (m)
*L*_*t*_	hydro turbine length (m)
*N*_*b*_	battery storage decision variable
Nt	number of hydraulic turbines
*P*_*b*_	available battery power (watt)
*P*_*dump*_	power dumped (unused)
*P*_*ESS*→*l*_	power supplied by ESS to load (watt)
*P*_*l*_	load demand (watt)
*P*_*losses*_	power incurred to cover RES losses (watt)
*P*_*m*_	power sent to motor unit of pump (watt)
*P*_*n*_	Available net power (watt)
*h*_*s*_	vertical distance between LR and UR (m)
*P*_*NS*_	deficit power not served (watt)
*P*_*outPV*_	solar array output power (kW)
*P*_*p*_	power available for pump(watt)
*P*_*pr*_	pump rated power (watt)
*P*_*pv*_	power produced by solar subsystem
*P*_*RE*→*l*_	power directly supplied by RE generators to load
*P*_*ti*_	net deficit power sent to hydro turbine unit (watt)
*P*_*to*_	hydro turbine power output (watt)
*P*_*tr*_	hydro turbine rated power (watt)
*P*_*wt*_	power produced by WT subsystem
*Q*_*p*_	pump flow rate (m^3^/sec)
*Q*_*t*_	hydro turbine flowrate (m^3^/sec)
*Q*_*tr*_	hydro turbine rated flowrate (m^3^/sec)
*Re*	reynold number
*R*	interest rate (%)
*SOC*_*b*_	battery storage SOC
*SOC*_*min*_	minimum SOC
*SOC*_*max*_	maximum SOC (100 %)
*T*_*amb*_	ambient temperature (^°^*C*)
*T*_*PV*_	PV module cell temperature (^°^*C*)
*T*_*STC*_	PV module cell temperature under standard test conditions(^°^*C*)
*t*_*vlo*_	turbine valve openness
*Μ*	dynamic viscosity of water (8.9 ∗ 10^-4^ pa.s)
*V*	water velocity (m/s)
*V*_*b*_	nominal battery voltage
*Y*_*PV*_	solar array rated power (kW)
*η*_*c*_	BS charging efficiency (%)
*η*_*d*_	BS discharging efficiency (%)
*η*_*m*_	motor efficiency (%)
*η*_*p*_	pump efficiency (%)
*η*_*t*_	hydro turbine efficiency (%)
∀_*b*_	BS energy capacity
∀_*ur*_	volume of water in the upper reservoir (m^3^)
∀_*urv*_	upper reservoir volume (m^3^)
*Ρ*	water density (997 kg/m^3^ at 25 οC)
*Ε*	absolute roughness (mm)
Acronyms	
BS	battery storage
COE	cost of energy
DSF	demand-supply fraction
EMS	energy management strategy
ESS	energy storage system
GWO	grey wolf optimizer
LR	lower reservoir
NPC	net present cost
PHS	pumped hydro storage
PV	solar photovoltaic
BS	battery storage
COE	cost of energy
DSF	demand-supply fraction
EMS	energy management strategy
ESS	energy storage system
GWO	grey wolf optimizer
LR	lower reservoir
NPC	net present cost
PHS	pumped hydro storage
PV	solar photovoltaic
RE	renewable energy
RES	renewable energy system
RSR	energy supplied by RE to energy supplied by system ratio
SC	storage cost
SDR	oversupply index (supply to demand ratio)
SOC	state of charge
UR	upper reservoir
WT	wind turbine

#### Input data

The input data, i.e., wind speed, solar irradiance and load demand, is presented in the supplemental information (see Section S2). The mathematical modelling of PV and wind energy generation is comprehensively discussed in the supplemental information (see Section S2 & (Javed et al., 2019b; Adaramola et al., 2014)).

#### System description

An off-grid RES is employed for the developed methodology. The proposed RES contains RE generators (solar and wind), ESS (PHS and BS), an inverter, and a charge controller. The charge controller's function is to regulate, maintain and implement the developed methodology and take care of the whole system, i.e., avoid system breakdown/blackouts and voltage stability. Besides, based on the fed EMS, the charge controller will drive the ESS seeing the available net energy, i.e., periods of energy surplus/deficit. Finally, it will be decided whether there is a need for charging/discharging the ESS or no ESS will be activated for that period. Frequent cyclic charging and discharging of ESS will occur, and RES will guarantee a sustainable power supply. The methodology is discussed in detail in the following subsections.

#### Battery-PHS model

This section contains each ESS component mathematical modeling details considering all types of operational and efficiency losses. The proposed mathematical model is an extended form of the model presented by Mousavi et al.(Mousavi et al., 2019). In the previous study, the PHS model is only presented and experimentally validated. Meanwhile, in this study, the battery model is integrated. Design parameters are optimized, and the developed model is embedded with a designed load to investigate the significance of the created model for defined technical and economic indicators. Furthermore, the proposed model is easy to replicate, and researchers can easily replicate the generated model for future studies.

#### Pump model

The flow rate of the pump (*Q*_*p*_) is a function of net surplus power provided by the charge controller. The motor converts the available electrical input power (*P*_*m*_) into mechanical pump power (*P*_*p*_) with efficiency *η*_*m*_. The motor efficiency *η*_*m*_ is also a function of available power obtained from the efficiency-power curve (provided by the manufacturers). The motor and pump efficiency curves can be easily found in the product manuals (see Section S6). The available pump power can be modelled as:

(Equation 1) $$P_{p}=P_{m}.\eta_{m}$$

(Equation 2) $$\eta_{m}=f\left(P_{m}\right)$$

*Q*_*p*_ is a function of mechanical power, available pump head (*h*_*p*_) and efficiency of the pump (*η*_*p*_). It is important to note that pump efficiency is also a function of the flowrate and will be attained from the efficiency-flowrate curve.

(Equation 3) $$Q_{p}=\frac{P_{p}.\eta_{p}}{\rho .g.h_{p}}$$

(Equation 4) $$\eta_{p}=f\left(Q_{p}\right)$$

(Equation 5) $$h_{p}=h_{a}+h_{pl}$$

In Equation 5, *h*_*a*_ is the active head and *h*_*pl*_ is the pump head loss that is explained in the penstock losses model.

#### Hydro turbine model

The proposed hydro turbine model calculates the flowrate (*Q*_*t*_) required to satisfy the net deficit power (*P*_*ti*_) indicated by the controller. This model starts with defining the level of openness of the turbine valve (*t*_*vlo*_) that is placed at the outlet of UR. The turbine flow rate using the Bernoulli equation (for detailed description, see Section S3.2) can be modelled as:

(Equation 6) $$t_{vlo}=\frac{P_{ti}}{P_{tr}}\times 0.14$$

(Equation 7) $$Q_{t}=t_{vlo}\times a_{t}\times \sqrt{2gh_{t}}$$

(Equation 8) $$h_{t}=h_{a}-h_{tl}$$

(Equation 9) $$P_{to}=Q_{t}.h_{t}.\rho .g.\eta_{t}$$

(Equation 10) $$\eta_{t}=f\left(Q_{t}\right)$$

where *P*_*tr*_ is the turbine rated power (w); *h*_*t*_ is the available turbine head (m); *h*_*tl*_ is the turbine head loss that is explained in the penstock losses model; and *η*_*t*_ is the hydro turbine efficiency that is a function of turbine flowrate, i.e., efficiency-flowrate curve. The value 0.14 is defined based on the employed hydro turbine rated flowrate and efficiency range, i.e., efficiency-flowrate curve. The employed pump and hydro turbine's technical specifications are provided in supplemental information (Table S4).

#### Reservoir model

**Figure 7:**
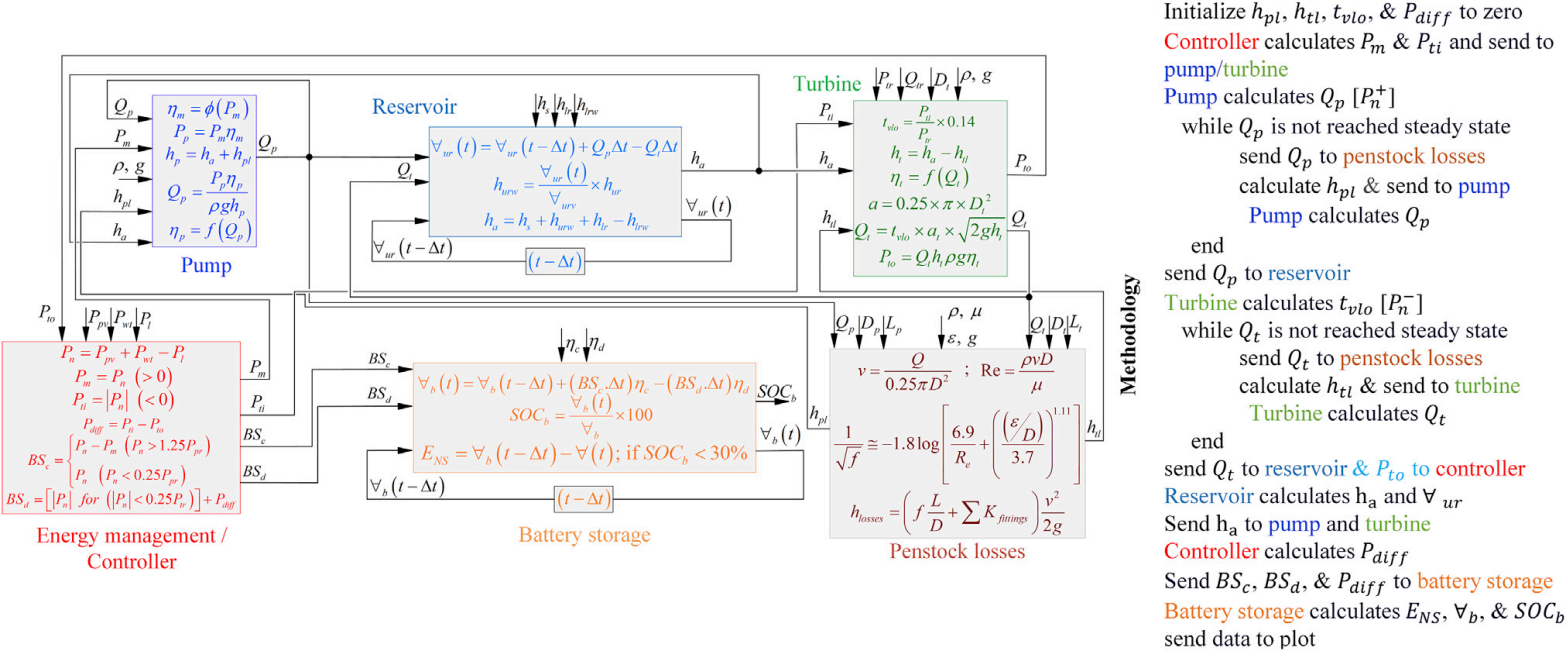
Developed hybrid storage mathematical model presentation with all involved losses All equations involved in the modeling of hybrid pump hydro battery storage are illustrated in this figure. Besides that, the mathematical model working mechanism is described for easy understanding. Full names of all involved parameters are provided in the nomenclature table.

Figure 7 illustrates each subsystem model of ESS with all the equations used and inputs needed to run the proposed model. A brief methodology is also described beside the figure that interprets the developed PHS-BS model's stepwise functioning. The reservoir model calculates the available water volume in the UR that is a function of incoming and outgoing flow.

(Equation 11) $$\forall_{ur}\left(t\right)=\forall_{ur}\left(t-\text{Δ}t\right)+Q_{p}.\text{Δ}t-Q_{t}.\text{Δ}t$$

Active head (*h*_*a*_) for the pump and hydro turbine can be calculated using the water level in the reservoirs.

(Equation 12) $$h_{a}=h_{s}+h_{urw}+h_{lr}-h_{lrw}$$

(Equation 13) $$h_{urw}=\frac{\forall_{ur}\left(t\right)}{\forall_{urv}}\times h_{ur}$$

The specifications of all head terms used in the mathematical modelling of PHS are described in supplemental information (Section S3.1). This model also considers the water level in the LR (*h*_*lrw*_) but depends on the incoming and outgoing flow from LR, geographical feature, and LR area, i.e., island, well, or small canal. If the LR water level does not change, the related terms can be ignored from the reservoir model.

#### Penstock losses model

The penstock losses model calculates the head losses (*h*_*pl*_,*h*_*tl*_) that are the function of pipe flowrate and fitting losses (The numerical value of fitting losses largely depends on the type of fitting, degree of openness, i.e., fully/partially open valves, and diameter to flowrate ratio. The specification of penstock employed in this study for the pump and the hydro turbine is given in the supplemental information [Table S13]) (Shammas and Wang, 2015).

(Equation 14) $$h_{losses}=\left(f\frac{L}{D}+\sum K_{fittings}\right).\frac{v^{2}}{2g}$$

where *f* is a friction factor and can be calculated using well known Colebrook equation (Application of Colebrook equation for the proposed PHS model is discussed in the supplemental information [Section S3.2]); *L* is the length of pipe; *D* is the pipe diameter; *K*_*fittings*_ is the resistance coefficient of fittings and *v* is the velocity of water. Figure 7 shows all the equations needed to calculate penstock losses as well as inputs and outputs of the model.

#### Battery storage model

Batter storage is used as supplementary storage in the proposed PHS-BS model to strengthen the off-grid RES reliability. Many literature studies have reported the complementary characteristics of BS and PHS(Ma et al., 2015; Javed et al., 2020). For instance, the battery has a high power density and response time that can be used to meet small surplus/deficit power. Meanwhile, it will reduce the start/stop numbers of pump/turbine and increase PHS machinery life, as PHS accounts for high storage capacity. In the proposed setup, BS will be derived by the controller based on the available net power and state of charge (SOC) of the BS. There will be continuous charging and discharging cycles of BS based on the defined maximum and minimum battery power (*P*_*b*_) constraints (For technical figures of employed battery, see supplemental information Table S5).

(Equation 15) $$P_{b}=\left\{ \begin{array}{l} P_{\min}=N_{b}.\min\left[0,\left(V_{b}.I_{b}.\left(SOC_{\min}-SOC_{b}\right)\right)\right]\\ P_{\max}=N_{b}.\max\left[0,\left(V_{b}.I_{b}.\left(SOC_{\max}-SOC_{b}\right)\right)\right] \end{array}\right.$$

where *N*_*b*_ is the optimization decision variable for BS. Available BS energy capacity and state of charge are modelled as:

(Equation 16) $$\forall_{b}\left(t\right)=\left\{ \begin{array}{l} \forall_{b}\left(t-\text{Δ}t\right)+\int_{t-1}^{t}BS_{c}.\eta_{c}.dt\;\left({P_{n}}^{+}\right)\\ \forall_{b}\left(t-\text{Δ}t\right)+\int_{t-1}^{t}BS_{d}.\eta_{d}.dt\;\left({P_{n}}^{-}\right) \end{array}\right.$$

(Equation 17) $$SOC_{b}=\frac{\forall_{b}\left(t\right)}{\forall_{b}}\times 100$$

#### Energy management strategy

Energy management strategy for off-grid RES has vital importance, especially when there are more than one dispatchable sources and a significant demand-supply mismatch. The mathematical models of solar and wind turbine subsystem are discussed in supplemental information (Section S2.1). The off-grid system's energy management is governed by the charge controller to regulate, improvise, and alarm during unusual situations, i.e., blackouts or failures. Figure 7 shows the required inputs to the controller and starts with calculating the available net power (*P*_*n*_).

(Equation 18) $$P_{n}=\left\{ \begin{array}{l} {P_{n}}^{+}\text{ for }P_{pv}+P_{wt}\text{>}P_{l}\\ {P_{n}}^{-}\text{ for }P_{pv}+P_{wt}<P_{l}\; \end{array}\right.$$

Positive net power $\left(P_{n}^{+}\right)$ will drive the ESS in charging mode and $\left(P_{n}^{-}\right)$ shows that there is a power deficit and additional power needed from ESS to meet the demand. During the periods of surplus and deficit power, the pump and turbine will be activated based on the allowable power range that can be presented as (Shabani et al., 2020; Javed et al., 2020):

(Equation 19) $$P_{m}=\left\{ \begin{array}{l} \max\left({P_{n}}^{+},0.25\times P_{pr}\right)\\ \min\left({P_{n}}^{+},1.25\times P_{pr}\right) \end{array}\right.$$

(Equation 20) $$P_{ti}=\left\{ \begin{array}{l} \max\left(\left|{P_{n}}^{-}\right|\;,\;0.25\times P_{tr}\right)\\ \min\left(\left|{P_{n}}^{-}\right|\text{ , }1.25\times P_{tr}\right) \end{array}\right.$$

The BS will cover the net power that the pump and hydro turbine did not cover due to the power range and SOC of the PHS. Moreover, besides the power range, the net power partially covered by the hydro turbine/pump (due to losses and SOC) will also be satisfied by the BS. If BS is unable to meet the power deficit due to low SOC, it will be considered as energy not served.

(Equation 21) $$BS_{c}=\left\{ \begin{array}{l} {P_{n}}^{+}-P_{m}\text{ for }\left({P_{n}}^{+}>1.25P_{pr}\right)\\ {P_{n}}^{+}\text{ for }\left({P_{n}}^{+}<0.25P_{pr}\right) \end{array}\right.$$

(Equation 22) $$BS_{d}=\left\{ \begin{array}{l} \left|{P_{n}}^{-}\right|-P_{to}\text{ for }\left(0.25P_{tr}\leq \left|{P_{n}}^{-}\right|\right)\\ \left|{P_{n}}^{-}\right|\text{ for }\left(\left|{P_{n}}^{-}\right|<0.25P_{tr}\right) \end{array}\right.$$

It is important to note that the controller will derive the BS as the primary storage for the period when the SOC of PHS is 100% or at a minimum level. Finally, the power balance of the whole RES can be modelled as:

(Equation 23) $$P_{l}+P_{m}+BS_{c}+P_{losses}+P_{dump}=P_{pv}+P_{wt}+P_{to}+BS_{d}$$

where *P*_*losses*_ refers to the power incurred to cover the whole RES losses, i.e., penstock and efficiency.

#### Multi-objective optimization

Many literature studies have recently used heuristic algorithms to optimize the capacity sizing of distributed RES(Khare et al., 2016). In this study, different multi-objective optimization cases are developed and optimized using the grey wolf optimizer (Mirjalili et al., 2014, 2016). A thorough discussion about the employed optimizer's structure and working mechanism in the multi-objective environment is presented in supplemental information (Section S4). Figure S6 briefly illustrates the functioning of the optimizer with the integration of the developed methodology (see also the attached Video S1: Demonstration of exploration and exploitation phases in multi-objective optimization). A multi-objective minimization problem can be defined as:

(Equation 24) $$\min f\left(x\right):\Gamma \to R^{k}=\left\{ \begin{array}{l} f_{1}\left(x\right):\Gamma \to R\\ ...\\ f_{k}\left(x\right):\Gamma \to R \end{array}\right.\text{ for }k\geq 2$$

where Γ→R represents the feasible region defined by the range of decision variable values and applied constraints, while *f*_1_ … *f*_*k*_ are the set of objective functions. Several sets of objective functions are optimized to evaluate the variation in their values with respect to each other, the relation between them and the optimal configuration size. Three technical and two economic objectives are considered. The technical objectives are the maximization of the demand-supply fraction (DSF), minimization of over-supply (SDR), and maximization of the ratio of energy supplied by RE to the energy provided by the RES (RSR); while economic objectives are minimization of cost of energy and storage cost.

## Supplemental information

Data S2. Optimization with three objectives, related to figure 2

Video S1. Demonstration of exploration and exploitation phases in multi-objective optimization, related to STAR METHODS

Video S2. Demonstration of multi-objective optimization simulation, related to figures 1 and 2

## Footnotes

Supplemental information can be found online at https://doi.org/10.1016/j.isci.2021.102730.
