# Supplementary material for: Quantifying techno-economic indicators' impact on isolated renewable energy systems
Source: iScience. 2021 Jun 12;24(7):102730. doi: 10.1016/j.isci.2021.102730 (PMC8258682; doi:10.1016/j.isci.2021.102730)
Supplement: Document S1. Figures S1–S9 and Tables S1–S7 [file mmc1.pdf]

iScience, Volume *24*

Supplemental information

Quantifying techno-economic indicators' impact on isolated renewable energy systems

Muhammad Shahzad Javed, Tao Ma, Navid Mousavi, Salman Ahmed, Henrik Lund, Hongxing Yang, and Yanjun Dai

Supplemental results

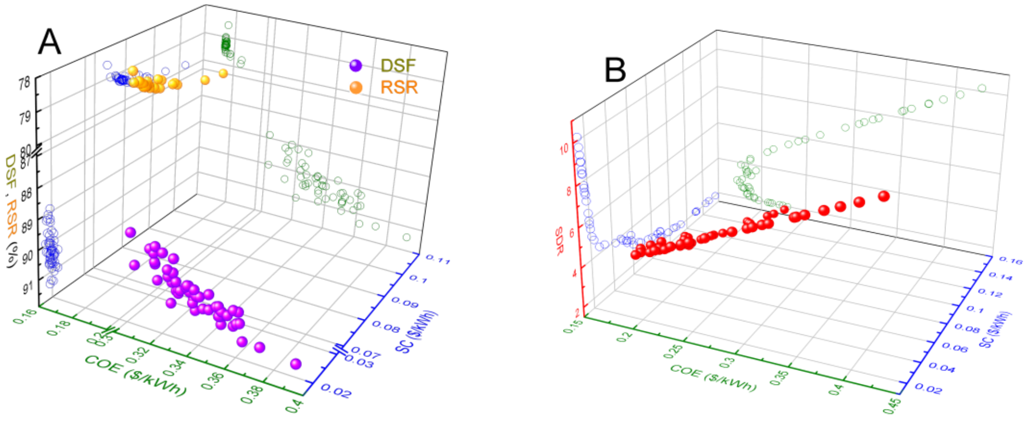

Figure S1. RES optimization with three objectives at reliability level of 90%. The description of the figure is presented with Figure 2 [Related to Figure 2].

Table S1. Specification of the configurations used for energy and cost-share analysis [Related to Figure 3].

	PV/WT	UR	Dp/Dt	BS	Pump/turbine	COE/SC	SDR/RSR
	(kW)	(m3)	(mm)	(kWh)	(W)	$/kWh	(ratio)
DSF = 95%	5.3/8.0	1010.6	91.5/90.5	3.9	3300/768	0.27/0.16	3.4/80.0
DSF = 90%	4.8/5.9	733.0	96.3/90.1	1.9	3300/768	0.22/0.13	2.9/81.8

S2 Mathematical modelling of system components

S2.1 Photovoltaic module

The solar PV array with 1 kW capacity is assumed in this study. The technical and cost details of the solar module are provided in Table S2. The relationship between incident irradiance and PV module output can be modelled as (Adaramola et al., 2014; Javed et al., 2019):

$$P_{outPV}(t) = Y_{PV} \cdot f_{PV} \cdot \left(\frac{G_{PV}(t)}{G_{STC}} \right) \cdot \left[1 + \alpha_{PV} \left(T_{PV} - T_{STC} \right) \right]$$

$$T_{PV}(t) = T_{amb} + \frac{G_{PV}(t) \times \left(T_{STC} - 20 \right)}{800}$$

where $P_{outPV}(t)$ is the output power of a PV array; Y_{PV} is the rated power of PV array (kW); f_{PV} is the solar module derating factor (%); $G_{PV}(t)$ is the incident irradiance (kW/m^2); G_{STC} is the standard irradiance (1 kW/m^2); α_{PV} is the temperature coefficient of power $\left(\%/^{\circ}C \right)$; T_{PV} is the PV cell temperature $\left(^{\circ}C \right)$; T_{STC} is the temperature of PV cell under standard test conditions $\left(^{\circ}C \right)$; and T_{amb} is the ambient temperature $\left(^{\circ}C \right)$. Sometimes, for normal temperature areas like islands, the PV module's surface temperature can be neglected and α_{PV} assumed zero (Adaramola et al., 2014). Therefore, PV output power equation can be simplified to;

$$P_{outPV}(t) = Y_{PV} \cdot f_{PV} \cdot \left(\frac{G_{PV}(t)}{G_{STC}} \right)$$

$$P_{PV}(t) = N_{PV} \times P_{outPV}(t)$$

where N_{PV} is the decision variable and will be optimized with the developed objective function and $P_{PV}(t)$ represents the total output power of the PV subsystem.

Table S2. Specification of solar subsystem [Related to STAR Methods] (Guezgouz et al., 2019; Javed et al., 2019).

Parameter	Value	unit
Model	polycrystalline	
Rated power	1	kW
Derating factor	80	%
Capital cost	896	$/kW
Operation and maintenance cost	15	$/kW-year
Lifetime	25	years
Efficiency	16.9	%

S2.2 Wind turbine

A 2 kW rated capacity WT is employed for this study. All WT specifications obtained from the manufacturer are provided in Table S3. Figure S2 illustrates the accuracy of the eight-degree polynomial equation used in this study to obtain the output power using available wind speed data.

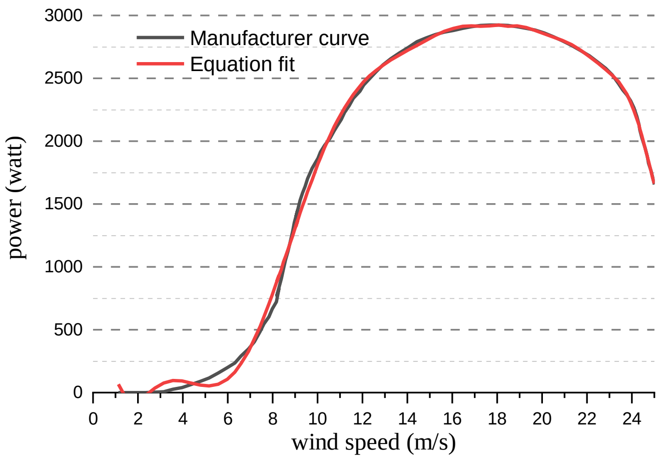

Figure S2. Wind turbine power curve used in study. The equivalent polynomial equation of the provided curve was made and employed in the model [Related to STAR Methods].

Table S3. Specification of wind turbine subsystem [Related to STAR Methods].

Parameter	Value	unit
Model	SW-2kW	
Rated power	2000	watt
Maximum power	2950	watt
Cut-in wind speed	2.5	m/s
Cut-off wind speed	25	m/s
Capital cost	998	$/kW
Operation and maintenance cost	20	$/kW-year
Lifetime	20	years

S2.3 Input data

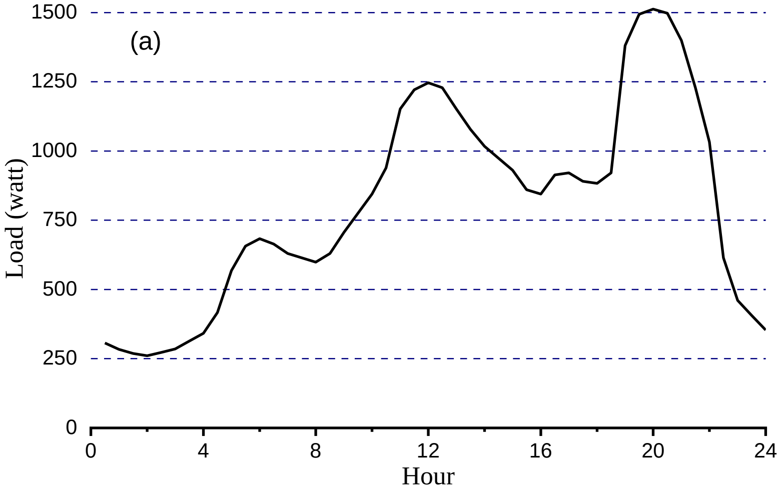

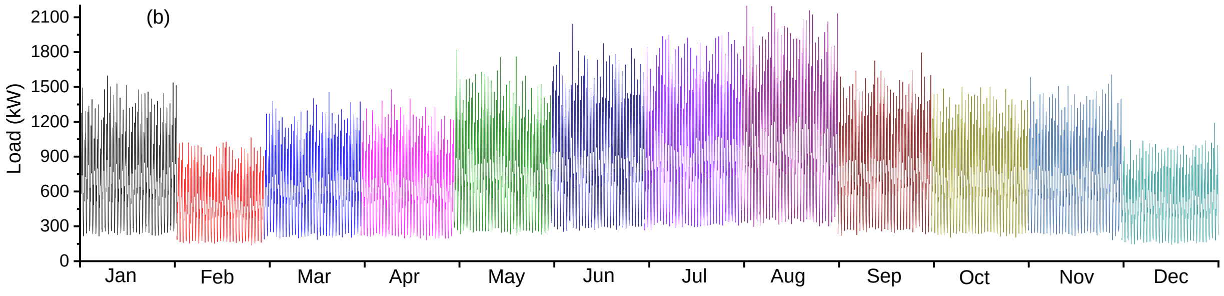

Figure S3. Designed load demand considering the daily and hourly randomness (a) Hourly load profile of a typical day (b) one-year load demand profile [Related to STAR Methods].

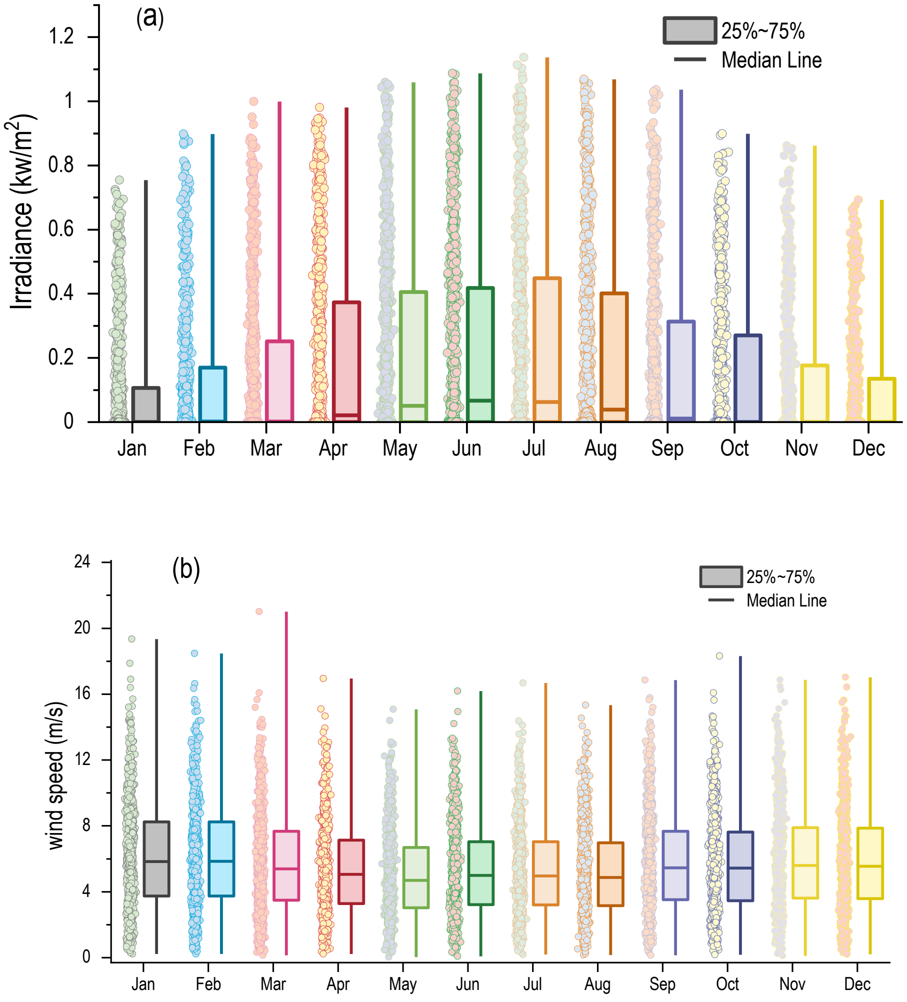

Figure S4. One-year renewable energy sources data used in this study (a) one year monthly solar irradiance (b) one year monthly wind velocity [Related to STAR Methods].

S3 Description of pumped hydro storage model

Table S4. Technical parameters of PHS components [*] [Related to STAR Methods].

Component	Parameters
Pump	Southern cross, type: MfD47A<br>Impeller diameter: 211mm, Motor: 3kW
Turbine	Power spout, Type: TRG<br>Rated: 768 watt, 15.3 liter/sec, 10m
Penstock	Material: carbon steel, Roughness (ε): 0.005,<br>Length: 12.5 m
Reservoir	Height: 2m, $h_s = 8.5$ m

S3.1 Specification of head terms

h_a total/net available head for pump/turbine

h_{lrw} available water height in the LR

h_{lr} total height of LR

h_s static vertical distance between reservoirs

h_{ur} total height of UR

h_{urw} available water height in UR

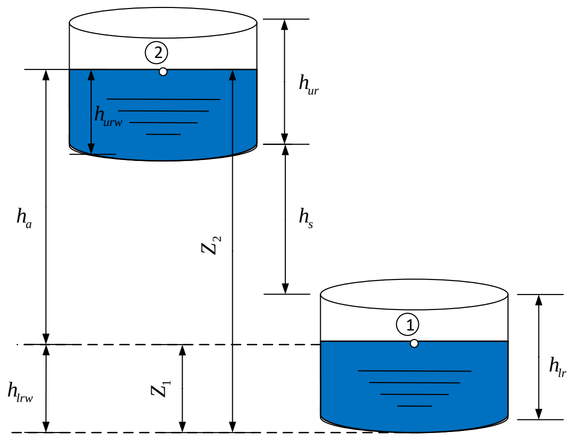

Figure S5. PHS reservoirs layout [Related to Figure 7].

[*] The technical specifications of the PHS components used in this study can be obtained from the authors for academic purpose.

S3.2 Calculation of hydro turbine flow rate

Bernoulli equation (Mousavi et al., 2019):

$$\frac{P_1}{\rho_1 g}+\frac{v_1^{\,2}}{2g}+h_1+h_{tl}=\frac{P_2}{\rho_2 g}+\frac{v_2^{\,2}}{2g}+h_2$$

It is important to note that hydro turbine output is considered as point 1 $(h_1=0)$ and point 2 is the water surface of the UR (Figure S5). Moreover, atmospheric pressure is assumed at point 2 and $v_2=0$. Meanwhile, the pressure at point 1 is a function of water height in LR. Therefore, Bernoulli equation can be simplified to:

$$h_2=h_{lrw}+\frac{v_1^{\,2}}{2g}+h_{tl}$$

Now, h_a for the turbine can be calculated as:

$$h_a=\frac{v_1^{\,2}}{2g}+h_{tl}=h_2-h_{lrw}\Leftrightarrow v_1=\sqrt{2g\left(h_a-h_{tl}\right)}\Leftrightarrow v_t=\sqrt{2gh_t}$$

where v_1 represents the hydro turbine water velocity $\left(v_1=v_t\right)$.

Colebrook Equation (Shammas and Wang, 2015):

$$\frac{1}{\sqrt{f}}\cong -1.8\log\left[\frac{6.9}{\mathrm{Re}}+\left(\frac{\varepsilon/D}{3.7}\right)^{1.11}\right]$$

$$\mathrm{Re}=\frac{\rho vD}{\mu}$$

$$v=\frac{Q}{0.25\times\pi\times D^2}$$

Table S5. Technical parameters of battery storage (Ma and Javed, 2019) [Related to STAR Methods].

Parameter	Value
Battery nominal voltage (V_b)	2 V
Battery nominal current (I_b)	1000 A
Maximum depth of discharge	70%
Roundtrip efficiency	86%
Lifetime throughput	3326 kW
Lifetime	5 years

S4 Grey wolf optimizer

Table S6. Pseudocode of multi-objective grey wolf optimizer [Related to STAR Methods].

algorithm: Multi-objective grey wolf optimizer*

input: Irradiance, wind and load data, cost and technical details of system components

GWO parameters: a = [2,0], *nVar* = 7, max *iter* = 100, Archive size = 50

initialization: population = $X_i\left(i=1,2,3,...,n\right)$, A, and C

Calculate objective function values for each population search agent

Archive the non-dominated solutions

Select the leaders $\left(\alpha,\beta,\delta\right)$ from archived solutions

while the end condition is not met

for $X_i\left(i=1,2,3,...,n\right)$

update the position

end for

update A and C

calculate the objective values with updated positions for $X_i\left(i=1,2,3,...,n\right)$

get non-dominated solutions and update the archive

if the archive is full (maximum size = 50)

omit current archive member using a grid mechanism and add a new solution

end if

if the added archived solution is located outside the hypercubes

update the grids to cover the new solution

end if

20: select the leaders (α, β, δ) from archived solutions

21: *t = t+1*

22: *end while*

23: return archive

---

[*]For more details of GWO, besides the given description, readers are also advised to consult the reference literature studies (Mirjalili et al., 2014; Mirjalili et al., 2016).

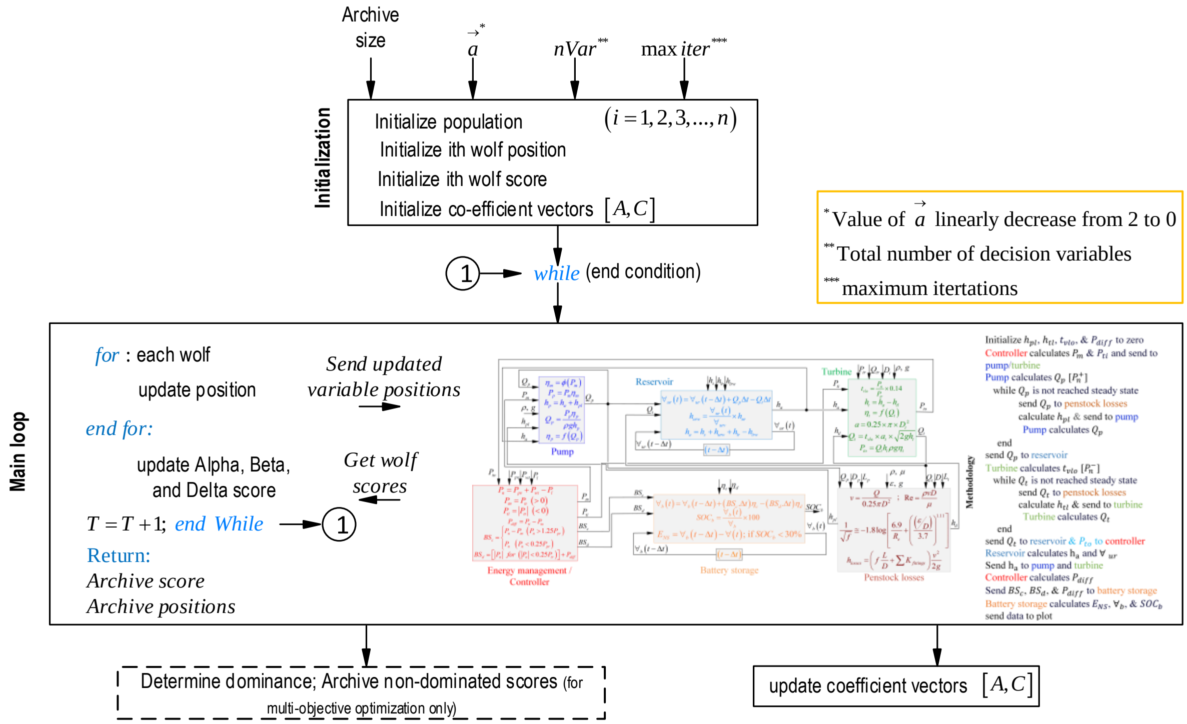

Figure S6. Application of gray wolf optimizer with developed energy system model [Related to Figure 7 & STAR Methods].

S5 Description of objectives

S5.1 Demand-supply fraction (DSF)

This objective reflects the reliability/autonomy of the RES. Each time, the optimizer will maximize its value by seeing the range of decision variables and constraints. A set of non-dominated solutions will be archived (due to the multi objectives).

$$DSF = \frac{H}{24 \times 365}$$

where H is the number of hours in a year during which the RES covered the demand.

S5.2 Oversupply index (SDR)

SDR is a ratio of the total energy generated/supplied by the RES to the energy demand met for a given period. It shows the amount of energy generated by RE generators not used to meet the load or stored in the ESS due to 100% SOC.

$$SDR = \frac{\sum \left(P_{RE \to l} + P_{ESS \to l} + P_{losses} + P_{dump} \right)}{\sum P_l - \sum P_{NS}}$$

S5.3 Renewable energy system self-sufficiency (RSR)

RSR is a ratio between the useful energy directly supplied by the RE generators to meet the load demand and the RES's total useful energy to satisfy the demand during a specific period. In other words, it is an index to check the mismatch between available RE sources and the demand of a particular place. It also shows the role of ESS in the RE environment, especially when RES is off-grid.

$$RSR = \frac{\sum P_{RE \to l}}{\sum P_{RE \to l} + \sum P_{ESS \to l}}$$

S5.4 Cost of energy (COE)

COE is a benchmark index for off-grid RES and has been extensively used in literature to assess the monetary benefits (Javed et al., 2021). COE estimates the cost of energy (in $/kWh) produced by the RES during a specific period (20 years for this study) and can be modelled as:

$$COE = \frac{\sum_{j} IC_j + \sum_{i=1}^{20} \frac{AC_j(i)}{(1+r)^i}}{\sum_{i=1}^{20} \frac{E_D(i) - E_{NS}(i)}{(1+r)^i}} \quad j = 1, 2, ..., \text{number of components}$$

where $E_D - E_{NS}$ is the net served energy. COE can be calculated by dividing the whole system cost, i.e., initial, replacement, operation and maintenance cost, with the systems's total useful energy.

S5.5 Storage cost (SC)

Storage cost is considered as an economic parameter in this study to analyze the significance of ESS in off-grid RES and its relation with other system evaluation indicators/objectives. SC of a RES can be modelled as:

$$SC = \frac{C_{ESS} \times S_{ESS}}{E_{ESS}}$$

where C_{ESS} is the storage capital cost (it includes all types of cost, i.e., maintenance and replacement); S_{ESS} is the storage capacity (kWh); and E_{ESS} is the total energy stored in the ESS during a given period.

Table S7. Economic parameters of the energy storage subsystem [Related to STAR Methods].

Component	Value*
Pump (3.3 kW) + pipe cost	388 $
PHS civil work + BOS cost	14 $/m^3
Hydro turbine (768 watt) + pipe cost	526 $

PHS O&M cost**	20 $/kW-year
BS cost	274 $/kWh
BS O&M cost	2 $/kWh-year
Inverter cost	336 $/kWh

*The cost values of ESS components are based on the Chinese market and information provided by the manufacturers.
**O&M cost refers to operation and maintenance cost.

S6 Pump and hydro turbine data provided by the manufacturers

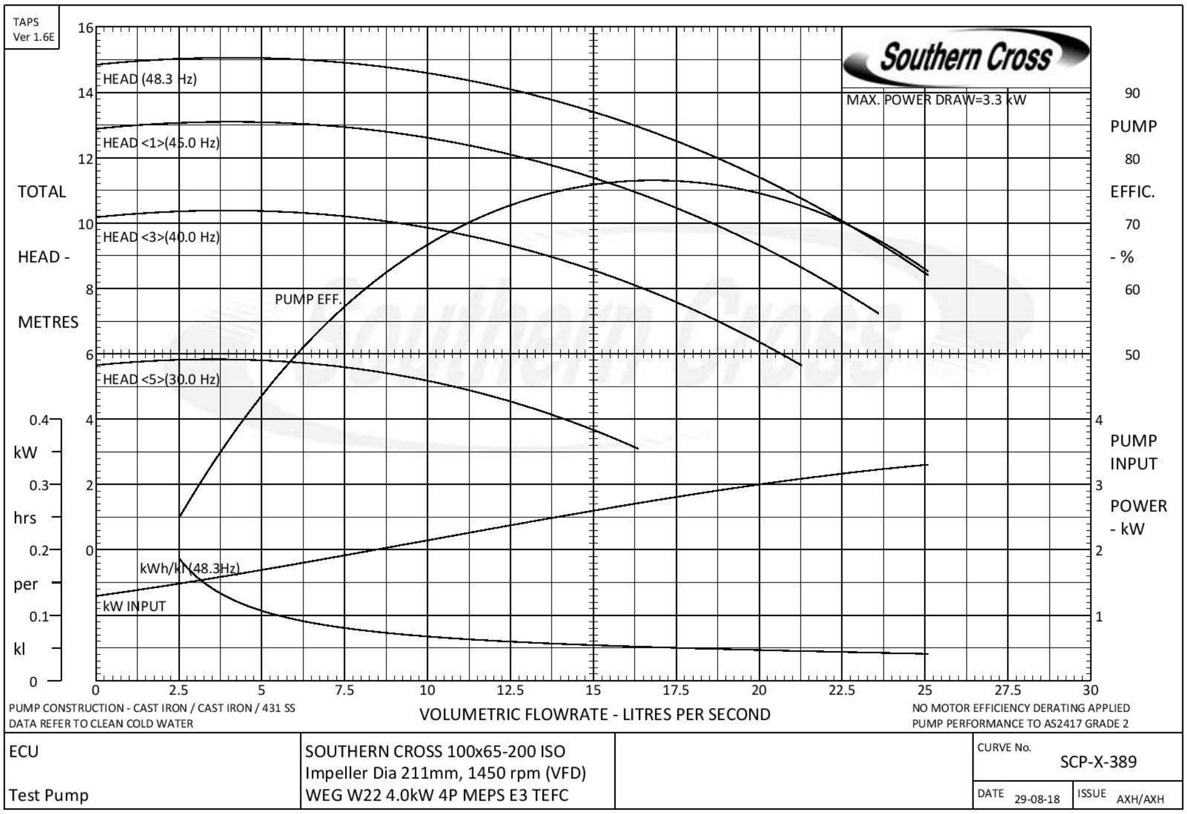

Figure S7. Pump flowrate-efficiency curve provided by the manufacturer [Related to STAR Methods].

CMG technology

Type Test Report

19 Corporate Avenue
Rowville 3178
Victoria Australia

Ph: 61 3 9237 4000
Testing@cmggroup.com.au
www.cmggroup.com.au

Test Details

TR Number: **P1631-1N**

Test Date: 12-Jun-01
Test Lab: NEPG Co Ltd

Name Plate Data

Three phase cage induction motor		
Type : PPA112M-4	Prod.Code: M34004003PPA	
4 kW	1445 r/m	Ser.No.: 1EWC40201
Duty: S1	cos fi: 0.88	50 Hz
415 V	Conn: Delta	7.2 A
IP: 66	Ins.Cl: H	Eff.[%] 87.7
Brg.DE: 6306-2Z	Brg.NDE: 6306-2Z	57 kg

Test Data

	Voltage [V]	Current [A]	Input Power [Watts]	Frequency [Hz]	Speed [r/m]	Torque [Nm]	Output Power [Watts]	Power Factor	Effic. [%]
No Load	415	3.5	210	50	1500	0.0	0	0.08	0.0
25% F.L.	415	3.2	1251	50	1488	6.7	1038	0.55	83.0
50% F.L.	415	4.3	2292	50	1476	13.0	2009	0.74	87.7
75% F.L.	415	5.7	3396	50	1462	19.6	3000	0.83	88.3
**100% F.L.**	**415**	**7.2**	**4560**	**50**	**1447**	**26.4**	**4000**	**0.88**	**87.7**
125% F.L.	415	8.9	5785	50	1430	33.4	4995	0.90	86.3
Locked Rt.T.	415	56.3	24810	50	0	71.32	-	0.61	-
Break D.T.	415	-	-	50	1149	82	-	-	-

Legend: F.L. = Full Load; Locked Rt.T. = Locked Rotor Torque Test; Break D.T. = Break Down Torque Test

**Resistance between terminals [Ohms]**	U1 - V1	2.3850
	U1 - W1	2.3840
	V1 - W1	2.3840
	Conn.	Delta
	@Temp[°C]	24.0
**Insulation resistance to frame**	Instrument	Megger
	voltage	500
	megohms	500
	@Temp[°C]	24.0
**H.V. test winding to frame**	Voltage	1830
	Minutes	1min.

**Heat Run Test**	Part of motor	Temperature rise in [K] at amb. temp. 21 [°C] measured by: thermometer	resistance	temp.detector
After continuous test at: 415 V, 7.4 A, 1447 r/m	Winding	-	45.2	-
	Frame	35.6	-	-
	Bearing	15.0	-	-

**Over rate tests**		Value	%incr.	Duration
	Excess Torque [Nm]	42.22	160	15sec.
	Excess Current [A]	10.81	150	2min.
	Overspeed [r/m]	1800	120	2min.

Measurement Accuracy

Current, Voltage, Power: +/-0.5%
Speed, Frequency: +/-0.5%
Resistance: +/-0.2%
Temperature: +/- 1 C

Exclusions/Deviations

Compliance to Standard

Motors tested in accordance with AS1359.101 Sections 7 & 8
Efficiency as per AS1359 Part 102.1

Comments

Tested By: Chen Yong Tang	Checked By: Zhou Yang Jia	Printed: 5-Nov-01

Figure S8. Pump motor data report provided by the manufacturer [Related to STAR Methods].

Water head (meter)	Water flow(liter/second)			
	5 liter/sec	6 liter/sec	7 liter/sec	8 liter/sec
10	150W	300W	350W	440W
11	165W	330W	385W	484W
12	180W	360W	420W	528W
13	195W	390W	455W	572W
14	210W	420W	490W	616W
15	225W	450W	525W	660W
16	240W	480W	560W	700W
17	255W	510W	595W	750W
18	270W	540W	630W	750W

Figure S9. Hydro turbine data provided by the manufacturer (output against available flow rate and head [Related to STAR Methods].
